# Inflammatory Environment Promotes the Adhesion of Tumor Cells to Brain Microvascular Endothelial Cells

**DOI:** 10.3389/fonc.2021.691771

**Published:** 2021-06-16

**Authors:** Ke Wang, Shuang Dong, Doaa Higazy, Lijing Jin, Qingcui Zou, Haowei Chen, Aakif Inayat, Sheng Hu, Min Cui

**Affiliations:** ^1^ State Key Laboratory of Agricultural Microbiology, College of Veterinary Medicine, Huazhong Agricultural University, Wuhan, China; ^2^ Key Laboratory of Preventive Veterinary Medicine in Hubei Province, The Cooperative Innovation Center for Sustainable Pig Production, Wuhan, China; ^3^ Key Laboratory of Development of Veterinary Diagnostic Products, Ministry of Agriculture of the People’s Republic of China, Wuhan, China; ^4^ International Research Center for Animal Disease, Ministry of Science and Technology of the People’s Republic of China, Wuhan, China; ^5^ Department of Medical Oncology, Hubei Cancer Hospital, Tongji Medical College, Huazhong University of Science and Technology, Wuhan, China; ^6^ Microbiology Department, Faculty of Agriculture, Cairo University, Giza, Egypt; ^7^ Hubei Provincial Cancer Center, Wuhan, China; ^8^ The Office of Hubei Provincial Cancer Prevention, Wuhan, China; ^9^ The Cancer Quality Control Center of Hubei Province, Wuhan, China; ^10^ College of Health Science, Huazhong Agricultural University, Wuhan, China

**Keywords:** tumor, brain metastasis, adhesion molecules, adhesion molecule ligands, human brain microvascular endothelial cells

## Abstract

Cancer patients usually suffer from unfavorable prognosis, particularly with the occurrence of brain metastasis of lung cancer. The key incident of brain metastasis initiation is crossing of blood-brain barrier (BBB) by cancer cells. Although preventing brain metastasis is a principal goal of cancer therapy, the cellular mechanisms and molecular regulators controlling the transmigration of cancer cells into the brain are still not clearly illustrated. We analyzed the mRNA expression profiles of metastatic brain tissues and TNF-α treated cancer cells to understand the changes in adhesion molecule expression during the tumor phase. To imitate the tumor microenvironment, an *in vitro* model was developed and the low or high metastatic potential lung tumor cells (A549 or H358) were cultured with the human brain microvascular endothelial cells (hBMECs) under TNF-α treatment. The analysis of online database indicated an altered expression for adhesion molecules and enrichment of their associated signaling pathways. TNF-α treatment activated hBMECs *via* up-regulating several adhesion molecules, including *ICAM1*, *CD112*, *CD47*, and *JAM-C*. Meanwhile, TNF-α induced an increased expression of adhesion molecule ligands such as *ALCAM* and *CD6* in both A549 and H358. Moreover, the expression of adhesion molecules and the ligands were also increased both in A549- or H358-hBMECs mixed culture system, which promoted tumor cells adhesion to endothelial cells. These results suggested that the enhanced interaction between tumor cells and brain microvascular endothelium might facilitate the incidence of metastatic brain tumors and further offer a better comprehension of brain metastasis prevention and treatment.

## Introduction

The metastatic tumor is a pathological change that migrated from the original site to new tissues or organs. Among malignant tumors, metastasis was responsible for about 90% of cancer deaths (1, 2), which indicated a serious public health concern around the world. However, brain metastasis accounts for about 25% of all the metastatic cancers (3). As an organism’s immune privilege area, the blood-brain barrier (BBB) restricts the passage of medicine or immune cells to the brain, which might be one reason of patients to deteriorate and even to death upon the occurrence of brain metastasis.

Solid tumor cells undergo a series of complicated processes to spread to the circulatory system (4). The adhesion of the circulating tumor cells to the BBB is a critical step of brain metastasis (5). The BBB and blood-cerebrospinal fluid barrier separate the central nervous system from the circulatory system. The BBB is structurally composed of endothelial cells, pericytes, astrocyte endfeet, and neurons, while the endothelial cell tight junctions combined with pericytes form the basic structure of BBB (6). The endothelial cells play an important role in maintaining the BBB, thus, the cell dysfunction always indicates a change of BBB permeability. Many diseases, such as neoplasia and viral infection, can induce the BBB breakdown (7, 8). However, brain metastasis often starts with endothelial cell activation. A recent study suggested that solid tumors changed the microenvironment and resulted in system inflammation (9). The induction of various inflammatory factors such as vascular endothelial growth factor C (VEGFC) could mediate endothelial cell activation during colorectal cancer invasion (10). Endothelial cell activation shows various changes, including the expression of adhesion molecules, cell morphologic alteration, cytokine production, and loss of barrier function. Previous studies indicated that inflammatory factors such as interleukin 8 (IL-8), tumor necrosis factor α (TNF-α), and VEGF mediated endothelial cell activation (11, 12), inducing the expression of adhesion molecules such as intercellular cell adhesion molecule 1 (ICAM-1), vascular endothelial cell adhesion molecule 1 (VCAM-1), and E-selectin, which were considered to promote leukocyte migration (13, 14). However, tumor cells may also spread to other organs under this mechanism.

Lung cancer is highly heterogeneous and known to spread to the brain in about 40%-50% of the metastatic cases, while breast cancer was about 15%~20% (15, 16). Tumor cells may create a chronic inflammatory microenvironment, producing various inflammatory factors such as CCL2, TNF-α, and S100A8/A9 (17–19). TNF-α stimulated the expression of cluster of differentiation 62E (CD62E, E-selectin) on human brain endothelium (hCMEC/D3) which affected the brain metastasis of non-small cell lung cancer *via* the interaction of CD15s with CD62E (20). Furthermore, the co-culture of human lung adenocarcinoma cell CL1-5 with human umbilical vein endothelial cells (HUVECs) altered the morphological structure of the latter (21). Thus, the inflammatory factors of the tumor microenvironment may be associated with the metastatic brain tumor.

The interaction of adhesion molecules and ligands is pivotal for tumor cells adherence to the endothelial cells, but the specific adhesion mechanism and the role of inflammatory factors in this process still need to be clarified. Through bioinformatics analysis, it was found that the adhesion molecules and their associated signaling pathways were up-regulated both in brain metastasis tumors and TNF-α stimulated cells. To verify these results, we used a mixed culture of lung tumor cells (A549 and H358) with brain microvascular endothelial cells (HBMECs) under TNF-α treatment to imitate the *in vivo* interaction. The A549 and H358 represented low and high metastatic potential lung tumor cells, respectively. A panel of genes associated with cell adhesion was screened with real-time PCR to assess the molecular event during the interaction systematically. The adhesion assay further indicated that the change of adhesion molecules and their ligands was associated with brain metastasis. These results may provide insights for preventing brain metastasis during tumor progression.

## Materials and Methods

### Publicly Available Data

The transcriptomic data of three different experiments uploaded to the Gene Expression Omnibus (GEO) database were studied. The first experiment concerned primary lung cancer from non-small cell lung cancer patients with or without brain metastasis. The second experiment specifies the expression array profiling data of human endothelial cells. Human umbilical vein endothelial cells (HUVECs) were treated with various Staphylococcus aureus or TNF-α (100 ng/ml, 3.5 h). Only the data from mock and TNF-α stimulated groups were analyzed in this study. The third experiment concluded the RNA sequencing data of TNF-α treated A549, and only the TNF-α treated (10 ng/ml, 6 h) or vehicle were extracted and considered in this research (22). The data for the three studies were downloaded from the GEO using the following accession numbers GSE126548, GSE82036, and GSM3538200, respectively.

### Cell Lines

Human lung adenocarcinoma A549 and non-small cell lung carcinoma H358 cells (American Type Culture Collection, Manassas, VA) were amplified and stored in the laboratory. Cells were maintained in Dulbecco modified Eagle medium (DMEM) containing 10% fetal bovine serum (Gibco, Grand Island, NY) and 1% penicillin-streptomycin (Beyotime, China). The human brain microvascular endothelial cells (hBMECs) isolated from the children′s cerebral microvessels were kindly provided by Prof. Xiangru Wang in the College of Veterinary Medicine, Huazhong Agricultural University, and originally obtained from John Hopkins University. hBMECs were cultured in RPMI 1640 medium supplemented with 10% fetal bovine serum, 1% penicillin-streptomycin, l-glutamine, sodium pyruvate, amino acid and vitamin in 37°C incubator with 5% CO_2_ and passaged every three days.

### Treatment of TNF-α on Various Cells

Confluent hBMECs, A549 or H358 were seeded on 6-well plates at a concentration of 5×10^5^ cells/ml. After overnight culture, hBMECs were stimulated with mouse recombinant TNF-α (R&D Systems, Minneapolis, MN, USA) at a concentration of 40 ng/ml, while A549 and H358 were stimulated with 5 ng/ml TNF-α. For the mixed culture system, 5×10^5^ cells/ml of hBMECs were seeded overnight, then 5×10^5^ cells/ml of A549 or H358 were added. The mixed cells were treated with DMEM medium containing 5 ng/ml TNF-α. After 24 h or 48 h, the supernatants were removed, and the cells were collected with TRIPure reagent (AidLab, China).

### Quantitative Real-Time PCR (qRT-PCR)

Total cell mRNA was extracted according to the manufacturer’s instructions of TRIPure reagent. Then the cDNA was synthesized with 5× All-In-One RT MasterMix (Applied Biological Materials, ABM, Canada). The qRT-PCR was performed with RealUniversal PreMix (TIANGEN, China) accordingly, and operated on a ViiA™ 7 Real-Time PCR System (Applied Biosystems, Foster City, CA). A series of adhesion molecules and adhesion molecule ligands were screened with qRT-PCR. The data was analyzed by the comparative CT method as previously described (23). The primer pairs used in this research are as follows: ICAM1 (5′-GCACATTGGTTGGCTATCTTCT-3′ and 5′-GCCCGAAGCGTTTACTTTGA-3′), ICAM3 (5′-CGAGTTCTTGCACAGGAACA-3′ and 5′-CCTGAAGACGTACATTAAGGCC -3′), JAM-A (5′-GTGCCTTCAGCAACTCTTCC-3′ and 5′-GAGCCGATATCCGTTTGGTC-3′), JAM-B (5′-GTCTCCTTTGTCTACTATCAAC-3′ and 5′-GGAGCCACTAATACTTCCAG-3′), JAM-C (5′-AAGGACGACTCTGGGCAGTA-3′ and 5′-CGCCAATGTTCAGGTCATAG-3′), ALCAM (5′-TCCTGCCGTCTGCTCTTCT-3′ and 5′-TTCTGAGGTACGTCAAGTCGG-3′), CD6 (5′-GTGACCTGAAGGAGAATCTGC-3′ and 5′-CCGGAGTGCAATCCTCTGG-3′), CD47 (5′-GGCAATGACGAAGGAGGTTA-3′ and 5′-ATCCGGTGGTATGGATGAGA-3′), CD99 (5′-AACCCACCCAAACCGATGC-3′ and 5′-TGAAAAGCTACCGGAGGAACTA-3′), CD112 (5′-GTCCTTCGTCTCTGCCAAGCA-3′ and 5′-CACTGCGTGGATGACCAGCTG-3′), SLC44A1 (5′-GGACCGTAGCTGCACAGAC-3′ and 5′-GCCACAAATAAATCCCATCCCA-3′), SIGLEC10 (5′-AAGGGACTCATCTCAACGGC-3′ and 5′-CCGTCTCTTCGGTAGAATCTTCA-3′), and ITGB2 (5′-TGCGTCCTCTCTCAGGAGTG-3′ and 5′-GGTCCATGATGTCGTCAGCC-3′).

### Cell Adhesion Analysis

The hBMECs were seeded on a 24-well plate. Then the cells were treated with TNF-α (40 ng/ml) or DMEM (control) for 24 h after the monolayers formed. Before adhesion assay, the A549 cells and H358 cells were suspended on PBS and labeled with CFSE tracer (Life Technologies, C34554, Eugene, USA) for 7 min of 3.75 μM, which was optimized according to manufacturer’s instructions. The cells were washed with DMEM, and the labeled cells were visualized with the inverted fluorescence microscope (EVOS Cell Imaging Systems, Life Technologies) at the excitation/emission of 488 nm/517 nm. Then 200 μl of A549 and H358 cells (containing 2×10^5^ cells) were incubated with hBMECs for 2 h. After three washes with PBS, the adherent tumor cells were observed and analyzed on the inverted fluorescence microscope.

### RNAseq Data Analysis

The downloaded RNAseq data were analyzed. The reads were confirmed to quality by FastQC, mapped, and assembled to the human reference genome GRCh38 (hg38) using HISAT2 and featureCounts, respectively (24). The DESeq2 R package was used to obtain the differentially expressed genes (DEGs), with a 5% *p*-adjusted value, and “apeglm” tool for log fold change shrinkage (25, 26). The R package clusterProfiler and DOSE were selected for the Gene ontology (GO) and Kyoto Encyclopedia of Genes and Genomes (KEGG) pathway analysis. While the figures were visualized using the R package enrichplot and ggplot2 (27).

### Statistical Analysis

The data are representative results from three independent experiments and shown as means ± SEMs. The significance was assessed with a two-tailed Student t-test or one-way ANOVA analysis, followed by Tukey’s *post hoc* tests with the GraphPad Prism software (v7.0; GraphPad, La Jolla, CA).

## Results

### Targeted Gene Expression of Brain Metastasis Accompanied Lung Cancer Cells

**Figure 1:**
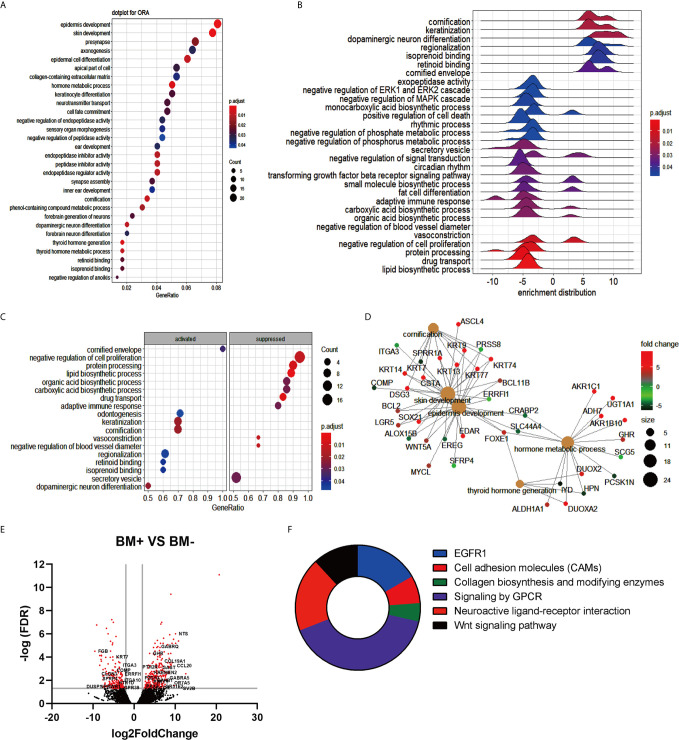
Transcriptome analysis summary of patients with brain metastasis (BM+) *vs* patients without brain metastasis (BM-). **(A)** Over representation analysis (ORA) for the enriched gene ontology (GO) terms obtained using the list of differentially expressed genes. The X-axis represented gene ratio, and the Y-axis represented GO terms organized upon the number of genes giving rise to each term indicated in the size of dots and adjusted p-value shown in the color range (Blue to Red) of the dots. **(B)** Ridge plot of enriched terms visualizing the expression distributions of core enriched genes for GSEA enriched categories. The positive and negative value refers to whether each term was up- or down-regulated. **(C)** Gene ontology based GSEA dot plot indicates the top 10 activated and suppressed GO terms and plotted in the order of gene ratio. The X-axis points to the gene ratio while the Y-axis specifies the enriched terms, and p-value is visualized in the dot color. **(D)** The cnetplot indicates the biological complexities among enriched GO terms and the overlapped genes in more than one term. The gray plots were the enriched pathways, while other plots represented genes. **(E)** Volcano plot exhibited the differentially expressed genes restricted to log2Foldchange (log2FC) of 1 and false discovery rate (FDR) ratio of 0.05. Red dots exhibit the significantly changed genes. **(F)** Pie chart of top 6 enriched GO terms that might be directly linked to the incidence of brain metastasis was presented.

To explore brain metastasis’s molecular events, RNA sequencing data were analyzed which were obtained from the GEO database for the primary tumor isolated from non-small cell lung cancer patients with brain metastasis (BM+) or without brain metastasis (BM-). The output results using the DESeq2 package indicated 488 differentially expressed genes (DEGs), among which 277 up-regulated genes and 211 down-regulated genes for the primary lung tumor cells of the BM+ patients over the BM- were observed. In the analysis, the DEGs were cut off to a log2FC of 2 and a p-value was adjusted to less than 0.05. The over-representation analysis (ORA) using clusterProfiler R package displayed the top 30 biological processes on a dotplot. The terms recording the highest gene ratio included the epidermic development (24/297, padj= 0.0007), epidermic cell differentiation (18/297, padj= 0.0083), skin development (23/297, padj= 0.0007), collagen-containing extracellular matrix (17/319, padj= 0.037), and keratinocyte differentiation (15/297, padj= 0.026) (**Figure 1A**). This result explains the active process of division, differentiation, and adhesion for lung cancer accompanied by brain metastasis. The gene expression distribution was observed with a ridge plot that elucidated a positive regulation of cornification, keratinization, and neuron differentiation. Negative modulation of signaling cascades, such as extracellular signal-regulated kinases (ERKs) and mitogen-activated protein kinase (MAPK) signal (**Figure 1B**), suggested the initiation of inflammation. Consistent with **Figures 1A, B**, however, the gene set enrichment analysis (GSEA) revealed the top activated and inhibited GO terms, in which cornification and keratinization were significantly activated while there was an obvious suppression regarding the negative regulation of cell proliferation and vessel diameter (**Figure 1C**). The linkage of genes and associated pathways indicated that cornification, skin development, epidermal development, hormone metabolic process, and thyroid hormone pathways are interconnected and overlapped among their functional genes. The up-regulated genes such as *DSG3* and *LGR5* might facilitate tumor metastasis, while the downregulation of *ITGA3* changed the extracellular matrix and might promote tumor expansion (**Figure 1D**). The volcano plot showed significant DEGs. In BM+ tumor cells *CCL20* exhibited a dramatic increase (**Figure 1E**), while cytokines/chemokines such as *CCL8*, *IL-6*, and tumor necrosis factor superfamily members also showed an increasing trend. There was also a substantial downregulation of extracellular matrix (*ITGA3* and *ITGA10*) and tight junction (*CLDN3*) transcripts (**Figure 1E**). Among the six biological processes of GO term enrichment, EGFR1, cell adhesion molecules, collagen, GPCR signaling, and Wnt signaling were possibly associated with tumor brain metastasis (**Figure 1F**). These results suggested that the change in cell division and cell differentiation might be associated with tumor progression. However, the ultimate suppression of the extracellular matrix and inflammation activation were probably responsible for tumor brain metastasis.

### TNF-α Induced the Expression of Adhesion Molecules and Adhesion Molecule Ligands on Human Endothelial Cells and Tumor Cells

**Figure 2:**
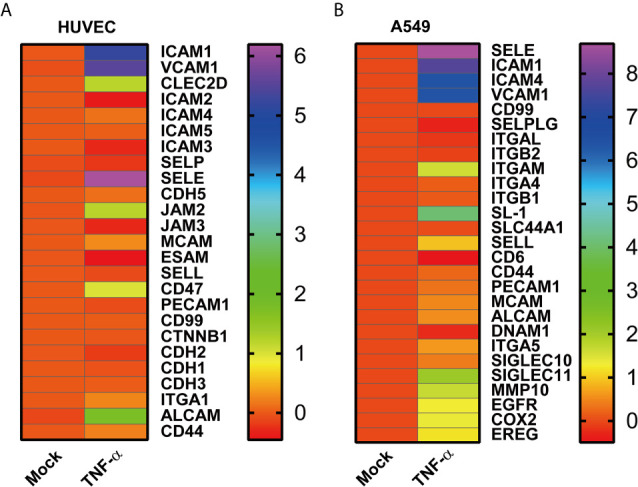
High throughput data analysis of HUVECs and A549 cells. **(A)** The heatmap indicates the mRNA adhesion molecules on HUVECs induced by TNF-α, and **(B)** adhesion associated genes on A549 determined in the presence of TNF-α. Each group (Mock and TNF-α) represented the average value of three duplicates. Data were shown as log2 fold change to mock.

To figure out the role of microenvironment inflammatory factor on endothelial cells and tumor cells, the data of TNF-α treated human umbilical vein endothelial cells (HUVECs) and TNF-α treated A549 data from the GEO database were analyzed. The TNF-α treated HUVECs induced the upregulation of adhesion molecules, including *ICAM1*, *VCAM1*, *CLEC2D*, *JAM2* (*JAM-B*), *CD47*, and *ALCAM* (**Figure 2A**). On the other hand, TNF-α stimulated A549 enhanced the mRNA expression of both adhesion molecules and adhesion molecule ligands such as *SELE*, *ICAM1*, *ICAM4*, *ITGAM*, *SL-1*, *SELL*, *SIGLEC10*, and *SIGLEC11* (**Figure 2B**). Moreover, TNF-α treatment modulated the expression of migration-related genes on A549, which included *MMP10*, *EGFR*, *COX2* and *EREG* (**Figure 2B**). It revealed that adhesion molecules and their ligands on endothelial cells and tumor cells induced by TNF-α might promote tumor adhesion.

### TNF-α Induced Human Brain Microvascular Endothelial Cells (hBMECs) Activation

**Figure 3:**
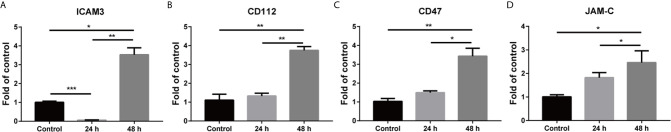
Activation of hBMECs after TNF-α treatment. Confluent hBMECs monolayers were treated with DMEM or DMEM containing 40 ng/ml TNF-α. Total RNA was collected at 24 h and 48 h. The quantification of cell adhesion molecule genes was detected with real-time PCR **(A–D)**. The fold change of treated cell adhesion molecule genes compared to the control over time was shown after normalized to β-actin. The data are representative of three independent experiments and expressed as mean ± SEMs. *p < 0.05; **p < 0.01; ***p < 0.001.

In vascular disease, numerous inflammatory cytokines and other factors are associated with endothelial cells activation (28). To identify whether cytokines could activate hBMECs, we applied a DMEM culture medium supplemented with 40 ng/ml TNF-α to the cultured cells. The mRNA levels of adhesion molecule genes were quantified with qRT-PCR. The expression of *ICAM3* was significantly decreased at 24 h but increased at 48 h compared to control after TNF-α treatment (**Figure 3A**). *CD112* (nectin-2) showed no changes at 24 h, but significantly up-regulated at 48 h (**Figure 3B**). However, the expression of *CD47* and *JAM-C* (junctional adhesion molecule C, also known as *JAM3*) began to elevate at 24 h and markedly increased at 48 h after TNF-α treatment (**Figures 3C, D**). These results suggested that variation in microenvironment mediated the activation of endothelial cells, and further exhibited a significant expression change of the adhesion molecules.

### Alteration of the Microenvironment Led to Upregulation of Adhesion Molecule Ligands on Tumor Cells

**Figure 4:**
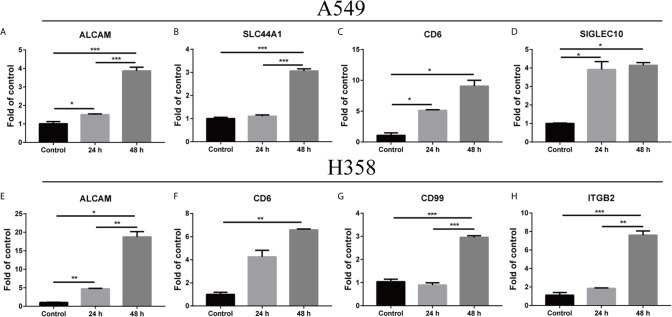
Change within the microenvironment induced the expression of adhesion molecule ligands on tumor cells. A549 and H358 cells were processed with TNF-α at 5 ng/ml concentration. Total RNA was extracted after 24 h and 48 h. The relative amounts of mRNAs expression were determined with real-time PCR **(A–H)**. The graph depicted the fold change of altered expression of the genes for treated cells compared to the control after normalized to β-actin. Data are shown as mean ± SEMs from three individual experiments. *p < 0.05; **p < 0.01; ***p < 0.001.

Inflammatory cytokines could induce cell-surface ICAM1 expression on tumor cells as previously described (29, 30). Human lung adenocarcinoma A549 cells were isolated from the solid tumors (31), while the non-small cell lung carcinoma H358 cells were originated from a metastatic tissue, the cells of which had low and high metastatic potential, respectively. Interaction of cell adhesion molecules and the ligands is a critical step of tumor metastasis (2). Hence, the expression of adhesion molecule ligands in A549 and H358 were detected in the presence of 5 ng/ml TNF-α. The mRNA level of *ALCAM* (activated leukocyte cell adhesion molecule), *CD6*, and *SIGLEC10* (sialic acid-binding Ig-like lectin 10) on A549 cells were substantially elevated at 24 h and peaked at 48 h compared to the control (**Figures 4A, C, D**). However, the *SLC44A1* (encoding CD92) dramatically increased on the mRNA level at 48 h but not at 24 h (**Figure 4B**). *ALCAM* expression on H358 markedly increased at 24 h and 48 h after treated with TNF-α, which was consistent with the result of *ALCAM* on A549 (**Figure 4E**). Similarly, the expression of *CD6*, *CD99* and *ITGB2* (encoding a subunit of LFA-1) on H358 cells showed no or slight change at 24 h but significantly up-regulated at 48 h compared with the control group (**Figures 4F–H**). These results indicated that inflammatory microenvironment could (such as TNF-α) mediate both low and high metastatic potential tumor cells expressing adhesion molecule ligands.

### Mixed Culture of A549 With hBMECs Induced the Expression of Adhesion Molecules and Adhesion Molecule Ligands in the Presence of TNF-α

**Figure 5:**
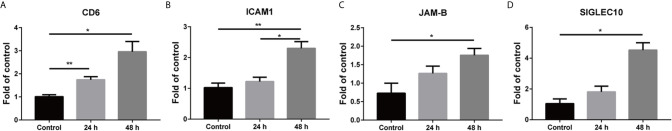
Mixed culture of A549 with hBMECs up-regulated the expression of adhesion molecules and adhesion molecule ligands. hBMECs were seeded overnight, and then A549 cells were added at a ratio of 1:1 under 5 ng/ml TNF-α treatment. Mixed cells were collected for RNA isolation at 24 h and 48 h. The altered gene expression of adhesion molecule and adhesion molecule ligand mRNAs were detected with real-time PCR **(A–D)**. The data are representative results from three independent experiment and displayed as mean ± SEMs. *p < 0.05; **p < 0.01.

To imitate the internal tumor migration environment, A549 cells and hBMECs were incubated on a culture medium supplemented with 5 ng/ml TNF-α. As receptors and ligands of adhesion molecules mutually interact, the expression of adhesion molecules and their ligands was determined on mixed cells. The expression of *CD6* was memorably elevated both at 24 h and 48 h after TNF-α treatment (**Figure 5A**). The mRNA of *ICAM1*, *JAM-B* (*JAM2*) and *SIGLEC10* showed no marked change at 24 h but significantly increased at 48 h compared to the control (**Figures 5B–D**). Taken together, it is suggested that TNF-α mediated the expression of adhesion molecules and their ligands after incubation of tumor cells with endothelial cells.

### Mixed Culture of H358 With hBMECs Mediated Adhesion Molecules and Adhesion Molecule Ligands mRNA Expression Under TNF-α Treatment

**Figure 6:**
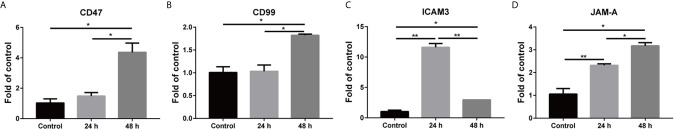
Mixed culture of H358 with hBMECs mediated the upregulation of adhesion molecules and adhesion molecule ligands. hBMECs were seeded overnight, and then H358 cells were added at a ratio of 1:1. The mixed cells were treated with 5 ng/ml TNF-α, and total RNA was extracted at 24 h and 48 h. Relative expression of mRNAs was determined with real-time PCR **(A–D)**. The data are representative results from three independent experiment and shown as mean ± SEMs. *p < 0.05; **p < 0.01.

To demonstrate whether high metastatic potential tumor cells could interact with endothelial cells, lung carcinoma H358 cells were incubated with hBMECs in a culture medium containing 5 ng/ml TNF-α. The expression of adhesion molecules and their ligands of mixed cells were quantified with real-time PCR. *CD47* and *CD99* only showed a substantial increase at mRNA level after TNF-α treated for 48 h (**Figures 6A, B**). Both *ICAM3* and *JAM-A* (*JAM1*) were dramatically elevated at 24 h and 48 h compared to control after treated with TNF-α (**Figures 6C, D**). Surprisingly, the *ICAM3* displayed a declining trend from 24 h to 48 h. These results, consistent with previous data, indicated that the microenvironment changed the adhesion molecules′ and their ligands′ expression during tumor-endothelial cell interaction.

### Microenvironment TNF-α Enhanced Adhesion of Tumor Cells to Endothelial Cells

**Figure 7:**
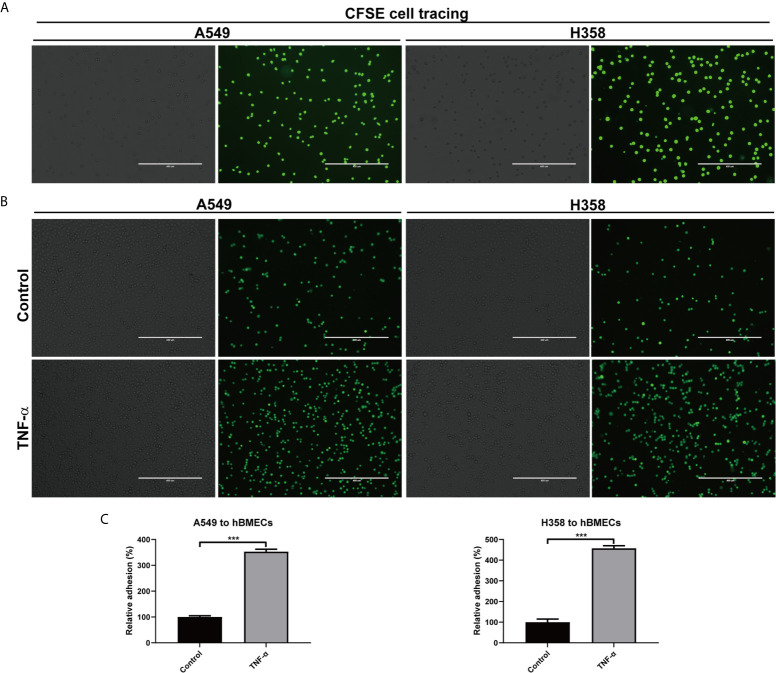
TNF-α promoted adhesion of tumor cells to endothelial cells. **(A)** A549 and H358 cells were labeled with CFSE dye, and the staining efficiency was evaluated with an inverted fluorescence Microscope. **(B)** Adhesion of A549 and H358 cells to hBMECs was observed on an inverted fluorescence Microscope after hBMECs treated with DMEM (control) or TNF-α (40 ng/ml). **(C)** The relative adherent cell numbers in **(B)** were quantified. One representative image of three independent experiments were shown in this figure. The left and right panels in each cell were the bright field image and fluorescent image, respectively. Scale bar was 400 μm. ***p < 0.001.

Since TNF-α up-regulated adhesion molecules and their ligands on tumor cells and endothelial cells, we wondered whether this inflammatory factor was associated with tumor cells adhesion. CFSE was used to trace the adhesion of A549 and H358 cells, which showed almost 100% labeling efficiency (**Figure 7A**). Adhesion assay demonstrated that both A549 and H358 could adhere to hBMECs to some extent (**Figure 7B**). Once stimulated with TNF-α, the adherent A549 and H358 cells increased dramatically, which were 253% and 357% (**Figures 7B, C**), respectively. By linking the current result with previous experiments, it is suggested that the overexpressed adhesion molecules and their ligands on endothelial cells and tumor cells by microenvironment TNF-α were responsible for tumor metastasis.

## Discussion

Tumor progression including growth, invasion, and metastasis, is closely related to the tumor microenvironment (32). The tumor microenvironment consists of non-tumor cells, such as endothelial cells, tumor-associated macrophages (TAMs), myeloid-derived suppressor cells (MDSCs), tumor-associated neutrophils (TANs), and various other cells, as well as cytokine interferon α (IFN-α), TNF, IL-1β and IL-6 to sustain an inflammatory environment (33). TAMs served as a potential anti-cancer therapeutic target because of its protumor functions (34). On the other hand, TAMs produced TNF-α both *in vivo* and *in vitro*, indicating that TAMs affected systemic inflammatory state (35). To mimic the *in vivo* tumor metastasis, a mixed culture model was utilized to incubate the tumor cells with hBMECs under TNF-α treatment. Obviously, there is an increased expression of adhesion molecules and their ligands in the mixed culture system with TNF-α treatment. It was speculated that these changes played a critical role in tumor brain metastasis during tumor progression.

Diverse humoral factors, including cytokines, lipopolysaccharides, polar phospholipids, homocysteine, and viral infection stimulated endothelial activation (14, 36–38). The activated cells were accompanied by adhesion molecules VCAM-1, ICAM-1 and E-selectin expression (39). IL-1α, IL-1β, IL-4 and TNF-α induced endothelial activation by mediating VCAM-1 production and promoting monocyte adhesion to endothelial monolayers, but nitric oxide limited this process (40). These studies suggested that endothelial activation was possibly involved in tumor brain metastasis. Indeed, TNF-α promoted adhesion of lung cancer cells to brain endothelium *via* a CD15s-CD62E manner, while blocking CD15s decreased this process (20). This result is consistent with our results. Moreover, the mixed culture of cancer cells with endothelial cells promoted angiogenesis and increased migration-related genes, indicating a boost of metastasis (21).

The interaction of adhesion molecules and adhesion molecule ligands widely existed during adhesion and migration of leukocytes. It was reported that ICAM-1- lymphocyte function-associated antigen 1 (LFA-1) interaction was essential for T or NK cells adhering to the target cells (41). ALCAM--CD6 mediated T cells adhering to dendritic cells and induced T cell proliferation (42). The specific interactions between adhesion molecules and their ligands were important for immune cell migration and activation. Similarly, the interaction of mutual adhesion molecules had multiple functions. ICAM-1-ICAM-1 homophilic interaction was associated with breast cancer metastatic amplification (43), while ALCAM-ALCAM interaction was an assistant for T cell adhering to dendritic cells (42). The interaction of identical adhesion molecules might become favorable in some pathological processes, such as tumor metastasis. In this study, we found an altered expression of adhesion molecules on endothelial cells and their ligands on tumor cells in the presence of TNF-α. This interaction might facilitate tumor-endotheliocyte adhesion, rolling, crossing, and contribute to tumor brain metastasis.

The specific mechanism of how tumor cells intruding into the central nervous system was ambiguous, but the current research could provide some cues. The possible routes of metastasis included the seed and soil hypothesis (44), the blood-brain barrier theory (45), and the tumor stem cell theory (46). However, the BBB theory was exquisite and much more attractive. Breast cancer cells infiltrated into the brain using a surface glycosylation α2,6-sialyltransferase ST6GALNAC5 to interact with and cross the BBB (47). Acute lymphoblastic leukemia cells (ALLs) invaded the central nervous system *via* crawling along the emissary vessels of subarachnoid space in an integrin-α6-laminin manner without breaching the BBB (48). JAM-A was believed to cause unfavorable prognosis in breast cancer patients *via* the ligand β1-integrin (49). However, Naik *et al*. demonstrated that JAM-A downregulation induced breast cancer cells to spread from the primary tumor site (50), which might due to the different tumor models and dynamic expression in different progression stages. The decrease of adhesion molecules induced tumor cells to disperse from the original site. However, upregulation of adhesion molecules enhanced tumor cells adhesion to endothelial cells and promoted tumor metastasis, which was generally presented in this study.

**Figure 8:**
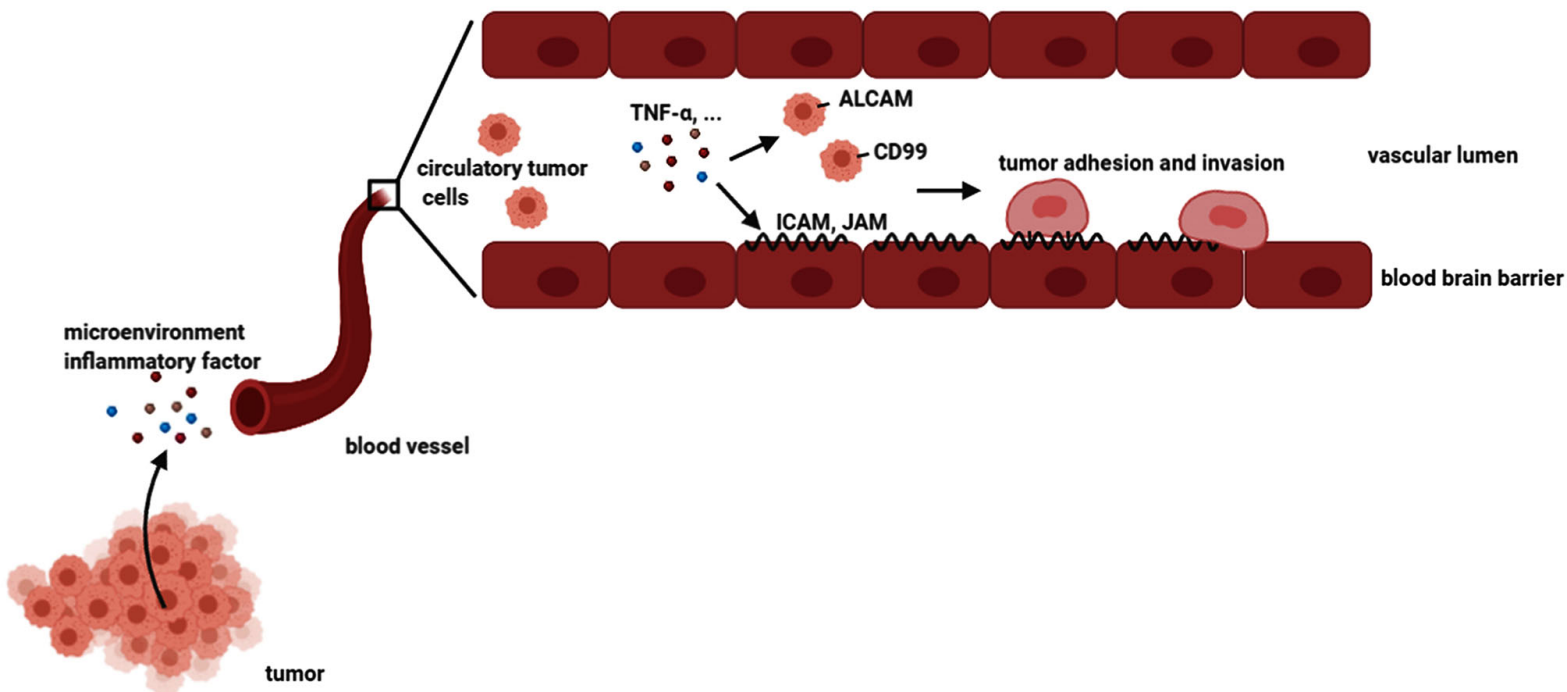
A schemata of tumor brain metastasis. The tumor cells could secrete many inflammatory factors. Some tumor cells could migrate to the circulatory system, which is called circulating tumor cells. Microenvironment inflammatory factors such as TNF-α could stimulate both brain endothelial cells and tumor cells, resulting in the upregulation of adhesion molecules and their ligands on endothelial cells and tumor cells, respectively. Consequently, the tumor cells adhered and invaded to endothelial cells, leading to brain metastasis.

These findings attracted our attention to the effect of adhesion molecules on tumor brain metastasis, as previously mentioned (5). As shown in **Figure 8**, our results suggested that the microenvironment TNF-α activated hBMECs endothelial cells, induced adhesion molecules expression, and mediated the expression of adhesion molecule ligands on tumor cells. It was indicated that TNF-α up-regulated adhesion molecules and their ligands in the mixed culture system and facilitated the interaction of tumor cells with endothelial cells that might further promote brain metastasis. Future research may focus on identifying the specific molecules that are associated with brain metastasis. Prospectively, a combination of inflammatory factors (such as TNF-α) and adhesion molecules (such as ICAM1 and ALCAM) neutralizing antibodies might be a useful adjuvant therapy to prevent tumor metastasis in the process of tumor treatment.

## Data Availability Statement

The datasets presented in this study can be found in online repositories. The names of the repository/repositories and accession number(s) can be found in the article/supplementary material.

## Author Contributions

Experiment was design by KW, MC, SD and LJ. The experiments of this study were performed by LJ, QZ and HC. The profile data were analyzed by DH, and the other data were analyzed by LJ, and KW. KW wrote this manuscript, while MC, AI, SD and SH kindly revised it. All authors have full access to this data and complete the responsibility part. All authors contributed to the article and approved the submitted version.

## Funding

The authors are grateful for the financial support provided by the National Program on Key Research Project of China (2016YFD0500406), the National Natural Sciences Foundation of China (Grant No. 31172294), the Fundamental Research Funds for the Central Universities Grant 2662018PY016, and Natural Science Foundation of Hubei Province (2019CFA010).

## Conflict of Interest

The authors declare that the research was conducted in the absence of any commercial or financial relationships that could be construed as a potential conflict of interest.

## Abbreviations

hBMECs, human brain microvascular endothelial cells; BBB, blood-brain barrier; TNF-α, tumor necrosis factor α; VEGF, vascular endothelial growth factor; ICAM-1, intercellular cell adhesion molecule 1; ALCAM, activated leukocyte cell adhesion molecule; JAM-A, junctional adhesion molecule A.
